# Cell-free DNA detected by “liquid biopsy” as a potential prognostic biomarker in early breast cancer

**DOI:** 10.18632/oncotarget.15120

**Published:** 2017-02-06

**Authors:** Roberta Maltoni, Valentina Casadio, Sara Ravaioli, Flavia Foca, Maria Maddalena Tumedei, Samanta Salvi, Filippo Martignano, Daniele Calistri, Andrea Rocca, Alessio Schirone, Dino Amadori, Sara Bravaccini

**Affiliations:** ^1^ Department of Medical Oncology, Istituto Scientifico Romagnolo per lo Studio e la Cura dei Tumori (IRST) IRCCS, Meldola, Italy; ^2^ Biosciences Laboratory, Istituto Scientifico Romagnolo per lo Studio e la Cura dei Tumori (IRST) IRCCS, Meldola, Italy; ^3^ Unit of Biostatistics and Clinical Trials, Istituto Scientifico Romagnolo per lo Studio e la Cura dei Tumori (IRST) IRCCS, Meldola, Italy

**Keywords:** CF-DNA, HER2, PI3KCA, prognosis, breast cancer subtypes

## Abstract

As conventional biomarkers for defining breast cancer (BC) subtypes are not always capable of predicting prognosis, search for new biomarkers which can be easily detected by liquid biopsy is ongoing. It has long been known that cell-free DNA (CF-DNA) could be a promising diagnostic and prognostic marker in different tumor types, although its prognostic value in BC is yet to be confirmed. This retrospective study evaluated the prognostic role of CF-DNA quantity and integrity of *HER2*, *MYC*, *BCAS1* and *PI3KCA*, which are frequently altered in BC. We collected 79 serum samples before surgery from women at first diagnosis of BC at Forlì Hospital (Italy) from 2002 to 2010. Twenty-one relapsed and 58 non-relapsed patients were matched by subtype and age. Blood samples were also collected from 10 healthy donors. All samples were analyzed by Real Time PCR for CF-DNA quantity and integrity of all oncogenes. Except for *MYC*, BC patients showed significantly higher median values of CF-DNA quantity (ng) than healthy controls, who had higher integrity and lower apoptotic index. A difference nearing statistical significance was observed for *HER2* short CF-DNA (*p* = 0.078, AUC value: 0.6305). *HER2* short CF-DNA showed an odds ratio of 1.39 for disease recurrence with *p* = 0.056 (95% CI 0.991-1.973). Our study suggests that CF-DNA detected as liquid biopsy could have great potential in clinical practice once demonstration of its clinical validity and utility has been provided by prospective studies with robust assays.

## INTRODUCTION

Breast cancer (BC) is the principal cause of cancer death in women worldwide.

As BC is a systemic disease at diagnosis, chemotherapy and hormonal therapy are usually given to eradicate any potential presence of occult micrometastasis after radical surgery, reducing the risk of relapse and improving overall survival according to validated prognostic factors [1].

Despite the locoregional and systemic treatment, 30% and 50% of the patients with negative and positive axillary lymph nodes, respectively, relapse after five years of surgery [1].

To date there are no recommended serum markers in clinical practice for monitoring patients and predicting their risk of relapse [2].

Further research is therefore needed to detect through liquid biopsy the biomarkers that can early identify patients at high risk of relapse and set appropriate systemic treatment, especially when clinical and instrumental evidence is not available.

Studies have long suggested that cell-free DNA (CF-DNA) could be used as a potential prognostic biomarker also for metastatic spread in solid tumors [3–6]. However, the lack of a standardized method for its evaluation remains one of its main critical issues.

Some authors have demonstrated that the concentration of circulating CF-DNA is higher in cancer patients than in healthy individuals [7–8].

DNA is released into the bloodstream by the apoptotic and necrotic cells of the primary tumor from an early phase of the disease, allowing for the extraction of DNA and the determination of the genetic and epigenetic characteristics [9]. It is well known that apoptotic cells release DNA fragments of 180–200 base pairs whereas necrotic cells release higher molecular weight DNA fragments [9]. It has been shown that DNA from normal apoptotic cells is highly fragmented, whereas DNA from necrotic cancer cells maintains its integrity. We evaluated the Apoptotic Index (AI) and the Integrity Index (II) as biomarkers to identify the patients likely to recur. The latter was calculated as the ratio of the quantity of long fragment/short fragment; the former was inversely calculated.

This retrospective study aimed to characterize CF-DNA as liquid biopsy for identifying patients at risk of relapse as a potential prognostic biomarker for all BC subtypes. We evaluated the role of some of the most frequently altered genes in BC (*HER2*, *MYC*, *BCAS1* and *PI3KCA*) in relation to patient prognosis due to a possible correlation with CF-DNA quantity and integrity.

## RESULTS

**Figure 1:**
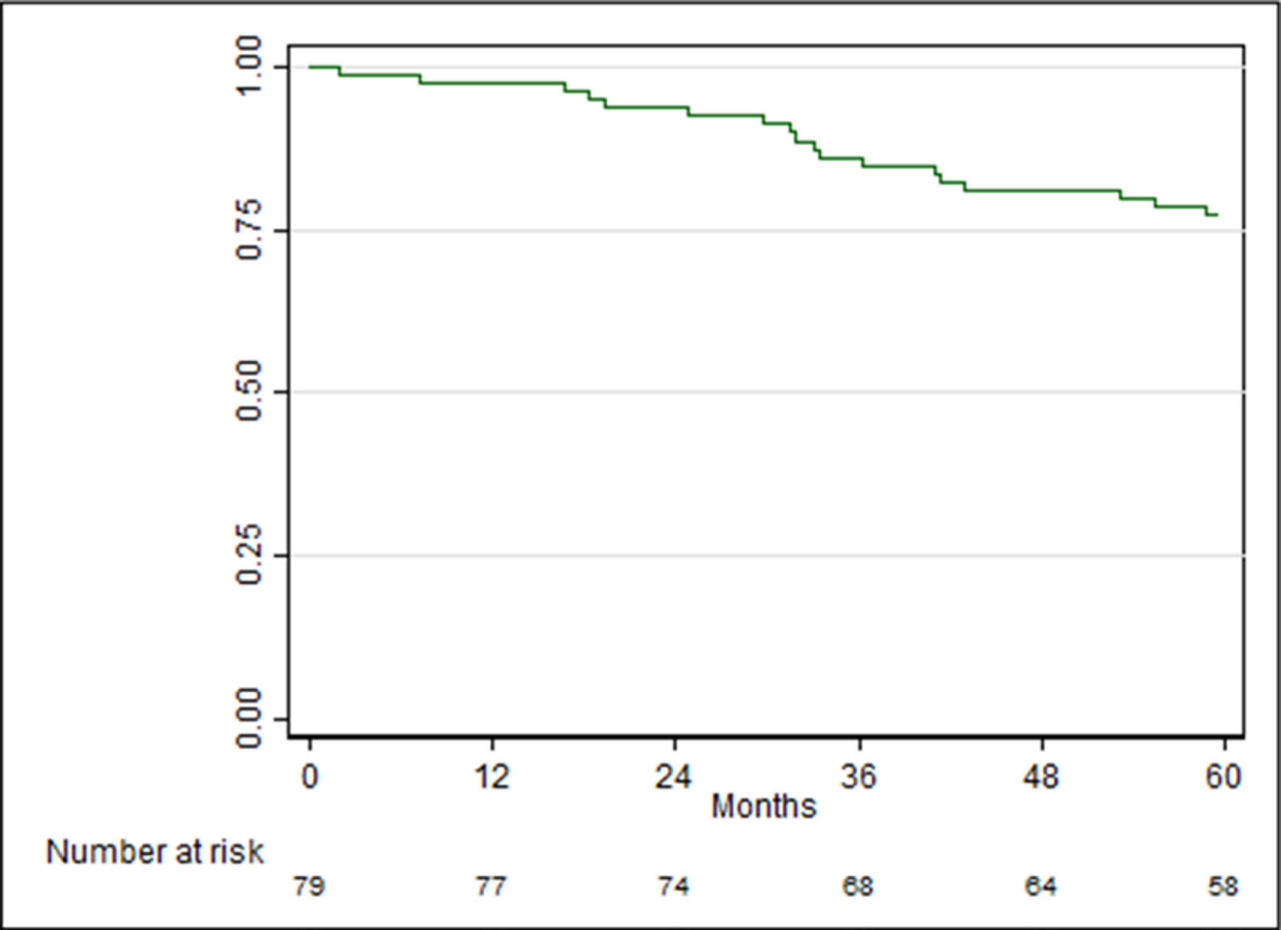
Recurrence-free survival (RFS) curve

Patients were matched by subtype and age. Median follow-up was 90 months (range 26–157). The recurrence free survival (RFS) curve showed that 77% (95% CI: 66%–85%) of patients was disease free at 5 years of surgery (Figure 1).

Clinical and histopathologic characteristics of patients in relation to prognosis are reported in Table 1.

**Table 1:** Patient clinic and histopathologic characteristics in relation to prognosis

Variable	Overall(*n* = 79)	Non-relapsed(*n* = 58)	Relapsed(*n* = 21)	*p*-value^#^
	Median (range)	Median (range)	Median (range)	
Age	57 (33–89)	57 (33–89)	59 (42–79)	0.698
	No. (%)	No. (%)	No. (%)	
Histological subtype				
A	23 (29.1)	17 (29.3)	6 (28.6)	0.984
B	21 (26.6)	16 (27.6)	5 (23.8)	
C	20 (25.3)	14 (24.1)	6 (28.5)	
D	15 (19.0)	11 (19.0)	4 (19.1)	
ER				
0	31 (39.2)	23 (39.7)	8 (38.1)	0.900
>0	48 (60.8)	35 (60.3)	13 (61.9)	
PGR				
0	40 (50.6)	29 (50.0)	11 (52.4)	0.852
> 0	39 (49.4)	29 (50.0)	10 (47.6)	
KI67				
0–19	39 (49.4)	29 (50.0)	10 (47.6)	0.852
≥ 20	40 (50.6)	29 (50.0)	11 (52.4)	
HER2 Status				
NEG	50 (64.9)	37 (64.9)	13 (65.0)	0.994
POS	27 (35.1)	20 (35.1)	7 (35.0)	
Grading				
G1	4 (5.5)	3 (5.4)	1 (5.9)	1.000
G2	27 (36.9)	21 (37.5)	6 (35.3)	
G3	42 (57.6)	32 (57.1)	10 (58.8)	
Therapy				
CT	27 (39.1)	19 (38.0)	8 (42.1)	0.353
HT	21 (30.4)	18 (36.0)	3 (15.8)	
CT + BT	14 (20.3)	9 (18.0)	5 (26.3)	
CT + HT +/– BT	7 (10.2)	4 (8.0)	3 (15.8)	

^#^Two-sample Wilcoxon rank-sum (Mann-Whitney) test for age, fisher exact test for histological subtype, grading and type of therapy and chi square for the remaining variables.

CT, chemotherapy; HT, hormonal therapy; BT, biological therapy.

No statistically significant difference was found in the extracted quantity of CF-DNA between the two categories of patients (non-relapsed and relapsed; median values, respectively: 9.465 ng - interquartile (iqr) range 5.530–12.700 vs 9.410 ng –iqr range 6.260–10.810, *p* = 0.673) and between healthy controls and patients (median values, respectively: 12.953 ng–iqr range 8.530–17.795 - vs 9.440 ng–iqr range 5.530–12.190, *p* = 0.078).

In addition to CF-DNA quantity analysis, the II and the AI were reported for each gene, calculated as the ratio of quantity of long fragment /short fragment (II) and short fragment /long fragment (AI).

CF-DNA quantity, II and AI were not statistically different between ER, PgR negative and positive tumors (data not shown). Also G1 and G2 tumors showed no statistically significant lower level of CF-DNA than undifferentiated BCs (data not shown).

Median values of the quantity (ng) of *HER2* short, *BCAS1* and *PI3KCA* short CF-DNA were significantly higher in BC patients than in healthy controls (Table 2). Healthy controls showed higher II and lower AI of *BCAS1*, *MYC* and *PI3KCA* oncogenes than BC patients (Table 3).

**Table 2:** CF-DNA quantity (ng) in relation to the status (cancer/control)

Gene	Overall(*n* = 89)	Cancer(*n* = 79)	Control(*n* = 10)	*p*-value^#^
	Median (iqr range)	Median (iqr range)	Median (iqr range)	
HER2 LONG	0.031 (0.012–0.121)	0.034 (0.011–0.137)	0.023 (0.015–0.033)	0.439
HER2 SHORT	0.202 (0.077–0.496)	0.238 (0.093–0.509)	0.067 (0.049–0.111)	0.016
BCAS1 LONG	0.241 (0.093–0.820)	0.266 (0.131–0.941)	0.039 (0.031–0.460)	0.021
BCAS1 SHORT	1.195 (0.476–2.069)	1.259 (0.536–2.133)	0.085 (0.050–0.916)	0.001
MYC LONG	0.421 (0.190–1.192)	0.421 (0.146–1.205)	0.426 (0.299–0.533)	0.658
MYC SHORT	1.124 (0.557–2.001)	1.145 (0.557–2.255)	0.771 (0.457–1.335)	0.193
PI3KCA LONG	0.089 (0.036–0.347)	0.108 (0.036–0.373)	0.056 (0.044–0.092)	0.398
PI3KCA SHORT	1.165 (0.231–3.434)	1.619 (0.296–4.332)	0.174 (0.140–0.390)	0.001

^#^Two-sample Wilcoxon rank-sum (Mann-Whitney) test.

**Table 3:** Integrity (II) and apoptotic (AI) indexes of each gene in relation to the status (cancer/control)

Index	Overall(*n* = 89)	Cancer(*n* = 79)	Control(*n* = 10)	*p*-value^#^
	Median (iqr range)	Median (iqr range)	Median (iqr range)	
II HER2	0.232 (0.080–0.440)	0.227 (0.057–0.449)	0.354 (0.229–0.436)	0.329
AI HER2	4.313 (2.274–12.567)	4.411 (2.226–17.612)	2.878 (2.293–4.370)	0.329
II BCAS1	0.296 (0.179–0.445)	0.286 (0.163–0.401)	0.520 (0.386–0.687)	0.002
AI BCAS1	3.379 (2.248–5.611)	3.501 (2.493–6.143)	1.925 (1.455–2.591)	0.002
II MYC	0.428 (0.229–0.660)	0.406 (0.210–0.636)	0.657 (0.428–0.914)	0.030
AI MYC	2.337 (1.515–4.375)	2.463 (1.572–4.766)	1.523 (1.094–2.337)	0.030
II PI3KCA	0.102 (0.055–0.286)	0.092 (0.051–0.190)	0.365 (0.240–0.806)	0.004
AI PI3KCA	9.759 (3.499–18.268)	10.852 (5.272–19.679)	2.759 (1.240–4.165)	0.004

^#^Two-sample Wilcoxon rank-sum (Mann-Whitney) test.

Except for *BCAS1* and *PI3KCA* short fragments, the analysis of CF-DNA quantity of each gene (long and short fragment) showed higher median values in relapsed patients than in non-relapsed patients although not statistically significant (Table 4). The indexes of each gene were the same in both relapsed and non-relapsed patients (Table 5).

**Table 4:** CF-DNA quantity (ng) in relation to patient prognosis

Gene	Overall(*n* = 79)	Non-relapsed(*n* = 58)	Relapsed(*n* = 21)	*p*-value^#^
	Median (iqr range)	Median (iqr range)	Median (iqr range)	
HER2 LONG	0.034 (0.011–0.137)	0.032 (0.014–0.121)	0.049 (0.007–0.137)	0.868
HER2 SHORT	0.238 (0.093–0.509)	0.206 (0.087–0.466)	0.409 (0.123–0.890)	0.078
BCAS1 LONG	0.266 (0.131–0.941)	0.238 (0.093–0.798)	0.292 (0.169–0.970)	0.339
BCAS1 SHORT	1.269 (0.536–2.133)	1.277 (0.525–2.275)	1.269 (0.677–2.069)	0.824
MYC LONG	0.421 (0.146–1.205)	0.364 (0.164–1.379)	0.485 (0.120–1.048)	0.965
MYC SHORT	1.145 (0.557–2.255)	1.136 (0.557–2.246)	1.185 (0.689–2.255)	0.689
PI3KCA LONG	0.108 (0.036–0.373)	0.082 (0.032–0.315)	0.195 (0.049–0.472)	0.226
PI3KCA SHORT	1.619 (0.296–4.332)	1.630 (0.251–3.198)	1.619 (0.338–4.365)	0.549

^#^Two-sample Wilcoxon rank-sum (Mann-Whitney) test.

**Table 5:** Integrity (II) and apoptotic (AI) indexes of each gene in relation to patient prognosis

Index	Overall(*n* = 79)	Non-relapsed(*n* = 58)	Relapsed(*n* = 21)	*p*-value^#^
	Median (iqr range)	Median (iqr range)	Median (iqr range)	
II HER2	0.227 (0.057–0.449)	0.227 (0.085–0.440)	0.188 (0.022–0.556)	0.351
AI HER2	4.411 (2.226–17.612)	4.411 (2.274–11.698)	5.332 (1.799–46.085)	0.351
II BCAS1	0.286 (0.163–0.401)	0.285 (0.137–0.396)	0.291 (0.242–0.429)	0.277
AI BCAS1	3.510 (2.493–6.143)	3.510 (2.525–7.320)	3.439 (2.329–4.133)	0.277
II MYC	0.406 (0.210–0.636)	0.410 (0.200–0.655)	0.375 (0.237–0.558)	0.534
AI MYC	2.463 (1.572–4.766)	2.441 (1.526–5.011)	2.902 (1.793–4.219)	0.534
II PI3KCA	0.092 (0.051–0.190)	0.083 (0.050–0.174)	0.118 (0.081–0.260)	0.120
AI PI3KCA	10.852 (5.272–19.679)	12.042 (5.757–19.931)	8.487 (3.851–12.327)	0.120

^#^Two-sample Wilcoxon rank-sum (Mann-Whitney) test.

The relation between quantity of *HER2* CF-DNA and *HER2* positive BCs (*HER2* FISH amplified and/or IHC positive with 3+ score) showed no significant difference (data not shown), neither *HER2* index differed between the two *HER2* types of BC (data not shown).

The ROC curve analysis of the 4 oncogenes was performed to assess the ability of each gene to predict relapse. *HER2* short and II *PI3KCA* showed AUC values of 0.631 (95% CI 0.476–0.785) and 0.615 (95% CI 0.475–0.755), respectively, showing the highest accuracy in predicting relapse. The AUC value of the two-marker combination in a logistic regression model was 0.627 (95% CI 0.472–0.783), not statistically different from the AUC value of *HER2* short alone (*p* = 0.609). In addition, univariate conditional logistic model of *HER2* short CF-DNA showed an odds ratio (OR) of 1.39 for disease recurrence, with *p* = 0.056 (95% CI 0.991–1.973).

## DISCUSSION

There is urgent need for markers detected as liquid biopsy able to identify patients at high risk of relapse or progression. Many studies have failed to demonstrate the use of circulating biomarkers in BC follow-up often due to non-reproducible methods and to the intrinsic biologic value of the marker considered [10].

The assessment of CF-DNA as liquid biopsy could have great potential in clinical practice. Prospective studies on NSCLC patients and tests for target therapies have demonstrated the clinical validity and utility of CF-DNA especially in this population [11]. Contrasting results have been reported on what to analyze in CF-DNA and how: different features have been studied in CF-DNA as biomarker, such as integrity, gene copy number variations, mutations [11–15].

Different subsets of patients, timing of sampling, and methodology make analysis of CF-DNA more difficult to standardize and validate. Moreover, high detection rates are not reproducible in early stages of cancer probably due to insufficient DNA quantity of low tumor burden [14, 15]. High false-positive results in benign lesions in some cohorts have produced discordant results on the specificity of CF-DNA detection at baseline [16, 17]. Not even all patients with progressive metastatic disease appear to release tumor-derived DNA into the bloodstream in quantities measurable with the current technologies, providing heterogeneous and inconsistent data on early BC and adjuvant setting [6].

Despite the limitations of the this study, among which are the retrospective design and the low number of samples, we demonstrated the feasibility of CF-DNA evaluation on serum samples stored for a long period of time. We analyzed only serum samples collected before surgery (baseline). Our results confirmed literature data [7, 8] as CF-DNA quantity was significantly higher in terms of quantity (ng) of *HER2* short, *BCAS1* and *PI3KCA* short in BC patients than in healthy controls.

Despite our expectations, healthy controls showed higher II and lower AI of *BCAS1*, *MYC* and *PI3KCA* oncogenes than BC patients, in accordance with Ellinger et al. [18]. The potential prognostic value of *HER2* and *PI3KCA* as oncogenes has already been described. In particular, Garcia-Murillas and colleagues demonstrated that PI3KCA mutations detected in circulating tumor DNA can be related to microscopic residual disease that may help identify patients likely to recur [19]. In addition, Page and colleagues demonstrated the existence of amplified HER2 in CF-DNA on HER2 positive BC patients [20].

Our principal aim was to evaluate the prognostic role of CF-DNA in all BC subtypes. Yet we did not observe any role in predicting either relapse or progression. These findings were in accordance with Garcia-Murillas et al, who found that a mutational analysis of CF-DNA at baseline was not significantly associated with disease-free survival and risk of relapse [19].

Although this study showed no association between CF-DNA and prognosis, it is nevertheless useful for planning new prospective studies that take into consideration the time of sampling (pre-surgery, post-surgery, after and during therapy, over a long-term follow-up) and the molecular alterations by liquid biopsy, such as copy number variations, mutations and integrity of other genes.

Collection time, type of biological specimens (serum vs plasma), CF-DNA alterations and an adequate method of detection need robust assays to prove their clinical validity and utility. Our study points out that the great potential of CF-DNA detected as liquid biopsy still awaits rigorous prospective studies to enable widespread clinical application.

## MATERIALS AND METHODS

### Case series

A total of 79 serum samples were collected before surgery from women at first diagnosis of BC at the Breast Unit of the Morgagni-Pierantoni Hospital (Forlì, Italy) from 2002 to 2010. Twenty-one relapsed and 58 non-relapsed patients (approximately 1:3 ratio) were matched by subtype and age. Blood samples were also collected from 10 healthy donors. Recurrent disease in patients was defined as local, regional and distant relapse occurring at over 6 months of surgery.

The study protocol was reviewed and approved by IRST and AVR (Area Vasta Romagna, Wide Romagna Catchment Area) Ethics Committee (approval number 1267) and written informed consent was provided by each patient.

The healthy control group, reporting neither previous disease nor cancer, was matched to the patients by gender and age. CF-DNA quantity and integrity analysis was performed blindly on all individuals and was evaluable for the entire case series.

From each patient, 5 ml whole blood was collected in tubes without anticoagulant prior to surgery and centrifuged at 2500 *g* for 15 min within 2 h of collection. The supernatants were transferred into cryovials and immediately stored at –80°C until use.

### CF-DNA analysis

DNA was extracted and purified from 500 μl serum by Qiamp DNA minikit (Qiagen, Milan, Italy) according to the manufacturer's instructions. At the same time, DNA was extracted from a human genomic control using the same kit. DNA was quantified by spectrophotometry (NanoDrop ND-1000; Celbio, Milan, Italy). Real Time PCR reactions were carried out by Rotor Gene 6000 detection system (Corbett Research, St. Neots, UK) using IQ SYBR Green (Bio-Rad, Milan, Italy). All samples were analyzed for CF-DNA quantity and integrity of *HER2*, *MYC*, *BCAS1* and *PI3KCA* oncogenes.

Sequences >260 bp (“long”), corresponding to 4 oncogenes, were analyzed in addition to shorter sequences (≤ 125 bp, “short”) of the same genes, corresponding to: *HER2* (locus 17q12), *MYC* (locus 8q24 and 21), *BCAS1* (locus 20q.13.2), *PI3KCA* (locus 3q26.3). Primer sequences and amplicons length are shown in Table 6.

**Table 6:** Primer sequences and amplicons length

Gene	Primer sequence	Fragment size (bp)
HER2 long	Fw-CCAGGGTGTTCCTCAGTTGT Rev-TCAGTATGGCCTCACCCTTC	295
HER2 short	Fw- CCAGGGTGTTCCTCAGTTGT Rev-GGAGTTCCTGCAGAGGACAG	126
MYC long	Fw TGGAGTAGGGACCGCATATC Rev-ACCCAACACCACGTCCTAAC	264
MYC short	Fw-GGCATTTAAATTTCGGCTCA Rev-AAAAGCCAAATGCCAACTT	128
BCAS1 long	Fw-GGGTCAGAGCTTCCTGTGAG Rev CGTTGTCCTGAAACAGAGCA	266
BCAS1 short	Fw-GGGTCAGAGCTTCCTGTGAG Rev-TATCATGCCTTGGAGAACCA	129
PI3KCA long	Fw-CTC CACGAC CAT CATCAGGT Rev- CGAAGGTCACAAAGTCGTCT	274
PI3KCA short	Fw-CTCCACGACCATCATCATCAGGT Rev-TGGTTATTAATGAGCCTCACGG	129

PCR conditions for the long fragments were as follows: 95°C for 3 min; 45 cycles at 94°C for 40 sec; 56°C for 40 sec; 72°C for 1 min.

PCR conditions for the short fragments were as follows: 95°C for 90 sec; 40 cycles at 95°C for 15 sec; 54°C for 45 sec.

All Real Time PCR reactions were performed in duplicate on 10 ng of each DNA sample. Various amounts of DNA from the human genomic control (0.01, 0.1, 1, 5, 10 and 20 ng) were also analyzed to draw a standard curve. CF-DNA value for each sample was obtained by Rotor Gene 6000 detection system software using standard curve interpolation. Analysis was repeated whenever the difference between duplicate samples was > 1 cycle threshold. Real Time experiments were performed independently in duplicate on the same 8 samples to test assay imprecision. The coefficients of variation were then calculated for *HER2*, *MYC*, *BCAS1* and *PI3KCA* long and short fragments.

The CF-DNA II was calculated as the ratio of quantity of long fragment/short fragment. The CF-DNA AI was 1/II.

### Statistical analysis

Frequency tables were performed for categorical variables. Continuous variables were presented using median and range or iqr range. The relationship between clinic-pathological factors and prognosis was analyzed using a non-parametric ranking statistic test. Normality of data distribution was tested using Shapiro-Wilk test: data distribution was not normal and non-parametric statistical tests were used (Wilcoxon Mann-Whitney and chi-square test) to evaluate difference between relapsed/non-relapsed patients and between BC patients and healthy controls.

To evaluate the most discriminant cut-off values between relapsed and non-relapsed patients we used the receiver operating characteristic (ROC) curve analysis. True positive rates (sensitivity) were plotted against false positive rates (1-specificity) for all classification points.

Conditional logistic regression model was carried out to fully account for the structure of the data matched by histological subtype and age. OR and their 95% CI for patient prognosis were reported to identify factors independently associated with relapse of disease.

RFS was calculated from time of surgical treatment to time of disease relapse or death. *p* < 0.05 was considered statistically significant. Statistical analyses were carried out with STATA (version 14.1 for Windows, Stata-Corp, College Station, TX, USA).
